# Radiolysis generates a complex organosynthetic chemical network

**DOI:** 10.1038/s41598-021-81293-6

**Published:** 2021-01-18

**Authors:** Zachary R. Adam, Albert C. Fahrenbach, Sofia M. Jacobson, Betul Kacar, Dmitry Yu. Zubarev

**Affiliations:** 1 Department of Planetary Sciences, University of Arizona, Tucson, AZ 85721 USA; 2 Department of Earth and Planetary Sciences, Harvard University, Cambridge, MA USA; 3 School of Chemistry, University of New South Wales, Sydney, NSW 2052 Australia; 4 Department of Molecular and Cellular Biology, University of Arizona, Tucson, AZ 85721 USA; 5 Department of Astronomy, University of Arizona, Tucson, AZ 85721 USA; 6 Earth-Life Science Institute, Tokyo Institute of Technology, Tokyo, Japan; 7 IBM Research-Almaden, 650 Harry Rd., San Jose, CA 95120 USA

**Keywords:** Ecological networks, Ecosystem ecology, Planetary science, Chemical biology, Networks and systems biology, Planetary science, Astrobiology, Geochemistry, Chemistry, Origin of life

## Abstract

The architectural features of cellular life and its ecologies at larger scales are built upon foundational networks of reactions between molecules that avoid a collapse to equilibrium. The search for life’s origins is, in some respects, a search for biotic network attributes in abiotic chemical systems. Radiation chemistry has long been employed to model prebiotic reaction networks, and here we report network-level analyses carried out on a compiled database of radiolysis reactions, acquired by the scientific community over decades of research. The resulting network shows robust connections between abundant geochemical reservoirs and the production of carboxylic acids, amino acids, and ribonucleotide precursors—the chemistry of which is predominantly dependent on radicals. Moreover, the network exhibits the following measurable attributes associated with biological systems: (1) the species connectivity histogram exhibits a heterogeneous (heavy-tailed) distribution, (2) overlapping families of closed-loop cycles, and (3) a hierarchical arrangement of chemical species with a bottom-heavy energy-size spectrum. The latter attribute is implicated with stability and entropy production in complex systems, notably in ecology where it is known as a trophic pyramid. Radiolysis is implicated as a driver of abiotic chemical organization and could provide insights about the complex and perhaps radical-dependent mechanisms associated with life’s origins.

## Introduction

The origins of life may be conceptualised as a chronology that occurred within a completely enclosed box. Initially, energy and molecules are present within the box as abiotic, geochemically plausible forms of photons, gases, liquids and rocks. After some period of time, the chemical composition of the box includes biomolecules, living cells, molecular waste products, waste heat and any remaining initial substrates and side products. The basic assumption is that life and its organizational properties emerged from the conditions and materials present within the box, and was not delivered from elsewhere. At what point in this chronology did life’s complex organizational attributes arise? Are there abiotic settings that exhibit ‘life-like’ behaviours or chemical relationships near the very beginning of this prebiotic chronology?

Radiation chemistry has long been employed to model prebiotic chemical reaction networks, starting with Calvin’s 1951 report that radiolysis of aqueous CO_2_ results in its reduction^1^. Radical chemistry is responsible for a large fraction of the products observed after radiolysis of dilute aqueous solutions containing various solutes. The relatively promiscuous nature of radicals, generated by radiolysis or other sources of energy, may be a possible means of driving the de novo emergence of chemical systems with complex attributes. Radicals (species with unpaired outer electrons) afford diverse sets of products starting from simple initial inputs and conditions, and can be generated by a variety of plausible mechanisms including ionizing radiation^2^, UV light^3^, spark discharge^4^ and mineral corrosion^5^, all of which have been implicated in proposed early Earth scenarios^6^. Radical reactions can proceed via low barriers without requiring further activation by a catalyst^7^. Radicals can exhibit powerful electrochemical potentials (far greater than even the most potent combinations of geochemical substrates^8^) and can be readily sourced from both organic and inorganic substrates to drive a wide array of organic redox reactions^9–11^. Indeed, a number of enzyme-facilitated biochemical redox potentials exceed tabulated geochemical potentials, indicating that non-enzymatic predecessors of these reactions, for kinetic reasons alone, may have required radiolysis, photolysis or extremely powerful phosphorylating intermediates to drive protometabolism^12^. For all of these reasons, radical-driven chemical networks may serve as insightful model systems for investigating the production of prebiotic molecules.

The chemical reactions that produce key reactive prebiotic molecules can be analysed at the network level much in the same ways as ecosystems and metabolic pathways^13–16^. A network is constructed by counting the object types (e.g., distinct molecules, atoms or photons) within a system and graphing each as a node; interactions (e.g., chemical reactions) between objects are depicted with lines, which are called edges. Mapped network interactions offer clues to how the laws of physics and chemistry can give rise to emergent features^17^. By describing the relationships between chemical objects that are synthesised alongside one another^18,19^, elements of hierarchical organization, feedback and order can be grasped—features that are missed at the level of studying individual reactions^20–22^. Through the analysis of reaction network maps, it is possible to assess whether an abiotic chemical system exhibits complex organizational attributes associated with life.

Living systems exhibit persistent network attributes that reflect a balance between generic operating principles (e.g., persistence, robustness or redundancy) and the functional constraints associated with exhibiting these principles (e.g., cost, resource limitation or transport efficiency)^23^. We focus on three such attributes that are also implicated with broader examples of complex systems behaviour—states of matter capable of dynamic response as a result of state-dependent feedback mechanisms that regulate internal relationships between objects^24–27^. As in many different examples of complex systems, biological objects interact with a heterogeneous distribution that appears as a gradually decreasing slope (i.e., a ‘heavy tail’) on a connectivity histogram^28,29^. An observable is heavy-tailed if the probability of observing extremely large values is more likely than it would be for an exponentially-distributed variable^30^. Biological objects are also formed and interconverted within a topology of overlapping cycles of energy and matter^31–34^. Cycles are critical relationships that afford dynamic attributes such as buffering against supply fluctuations^35^, persistence^36,37^ and homeostatic maintenance^38,39^. Finally, biological objects exist within a nested hierarchy^40,41^ in which the production of numerous, smaller-sized objects supports the production of fewer, larger-sized objects. This arrangement is also known as a bottom-heavy energy-size spectrum^42^ which in the field of ecosystem ecology is more commonly referred to as a trophic pyramid. Bottom-heavy population distributions are intrinsically correlated with energy flow and entropy production across hierarchical tiers^42,43^, as objects at each level are inefficiently cycled through formation and degradation in closed loops to maintain far-from-equilibrium configurations^44–47^.

It was recently demonstrated that radiolysis, by means of a continuous reaction network, generates a complex mixture of products that includes precursors for nucleotide synthesis^48^. These findings add to a growing body of evidence that radicals provide particularly efficient means of connecting geochemical substrates to macromolecular precursors with minimal experimental intervention^2,12,49–54^. Here we analyse the structure of a reaction network compiled from hundreds of experiments conducted over 60 years of research that links common geochemical substrates (CO_2_, H_2_O, N_2_, NaCl, chlorapatite and pyrite) to radiolytically-produced prebiotic precursors, and compare this network to life and its ecologies^55–57^.

## Results

A chemical reaction network of solid-, liquid-, and gas-phase reactions was assembled from 44 peer-reviewed publications spanning 31 journals over a period of time from 1961 to 2020 (Datafile S1). The network includes 782 reactions and 386 distinct atomic, molecular, and photonic species. To aid visualisation of the network, reactions were assigned to chemical reaction categories based on their reactive substrates (e.g., free radical reactions, physicochemical reactions, nitrile radical reactions, chloride reactions, geochemical reactions, etc.). Atoms are initially present in the system as their most geochemically abundant molecular species (H_2_O, CO_2_, N_2_, NaCl, a proxy for the apatite mineral group Ca_10_Cl_2_(PO_4_)_6_, and pyrite FeS_2_). The electromagnetic spectrum is binned into gamma, X-ray, UV, visible and infrared photons to account for reactions across key atomic and molecular energetic thresholds (i.e., strong nuclear binding force, inner valence electrons, outer valence electrons and ambient system temperature). Radiolytic energy was incorporated from natural modes of decay of radiogenic uranium oxide, which serves as a generic geochemical proxy for naturally-occurring radiolytic energy sources such as galactic cosmic radiation^58^, solar flares^59^ and planetary radiation belts^60^. A complete list of these reactions and source manuscripts is provided in Supplementary Datafile S1 (Excel spreadsheet) and Datafile S2 (.graphml network file).

**Figure 1:**
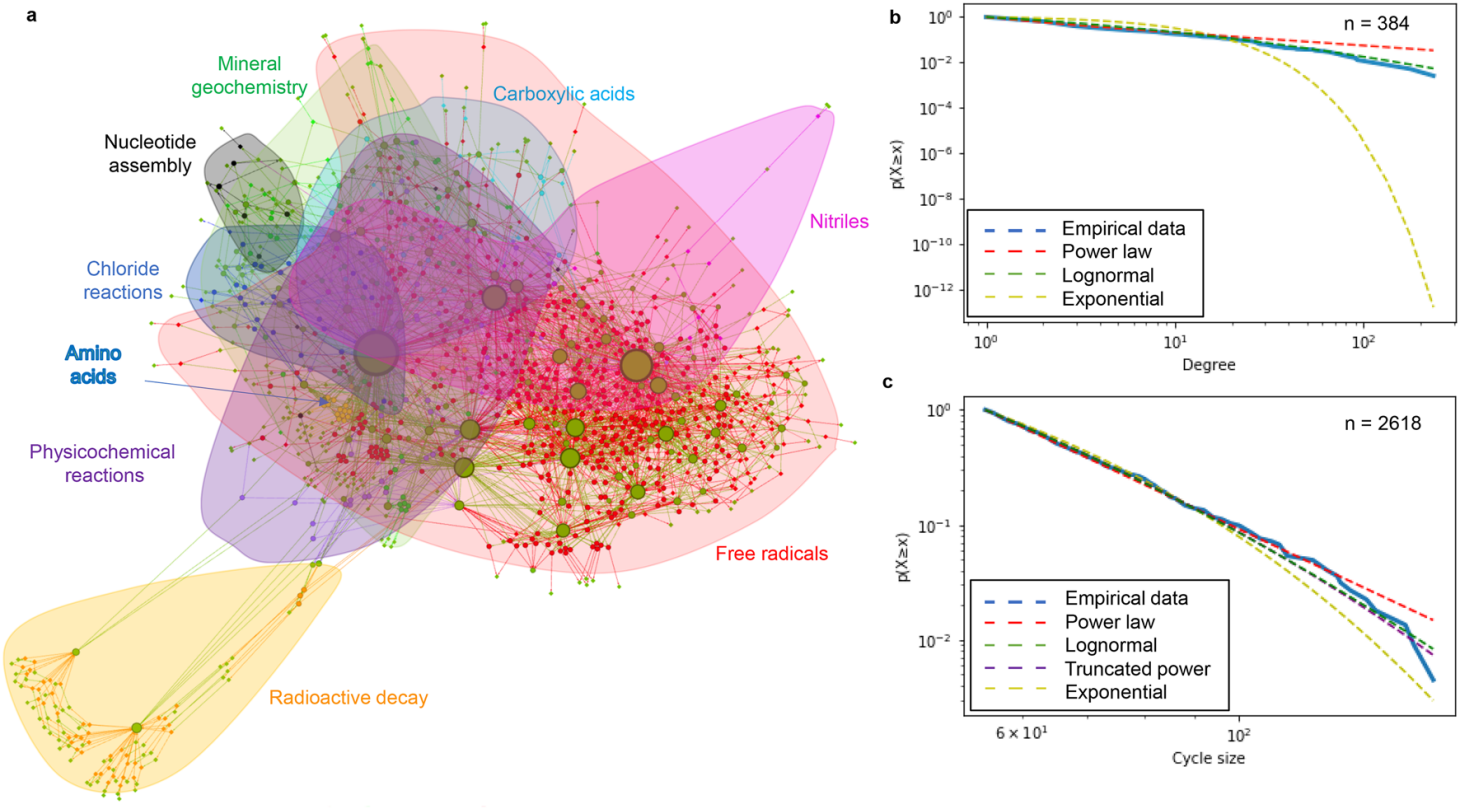
Visualisation and statistical analysis of radiolysis network. (**a**) Bi-partite network with molecular species (green circles) size-weighted according to degree of connectivity to reactions (red circles). Groupings of chemical reaction categories are individually shaded and labelled. (**b**) Statistical comparison of species node degree connectivity distribution indicates that an exponential fall-off fit is poor compared to a power law or lognormal fit of the empirical data. (**c**) Statistical comparison of the cycle size distribution indicates that an exponential fall-off fit is not reliably distinguishable from a power law, lognormal or exponentially truncated power law fit of the empirical data.

The radiolysis network is depicted in Fig. 1a using a ForceAtlas2 layout. Species (green) have radii weighted according to the total number of connections. The network has a diameter of 28, with an average directed path length of about 5.4 steps. Radical reactions (shaded in red) contain hubs that dominate the network and link together inputs and outputs from each of the reaction categories. The most connected network hub is water (234 total degree connections; Supplementary Table S1), but the next largest hubs are the low-mass, highly reactive reducing H (165 connections) and oxidising OH (122 connections) radicals, which are readily produced by radiolysis or photolysis of water. The most powerful forms of energy in the system (gamma rays, alpha particles, beta particles, X rays and UV light) are also major hubs within the network. Carboxylic acids are produced via radical reactions^53^. Amino acids are produced through a combination of radiolysis and acid-hydrolysis of uncharacterised polymers^2,61^. Nucleotide precursors that lead to cytidine and uridine cyclic phosphates are all present within the network^48,49,62^.

### Heavy-tailed connectivity distribution

A list of the most connected chemical species is provided in Supplementary Table S1. Scaling analysis and, specifically, evaluation of the relevance of the power law scaling, were conducted as described in the “Methods” section. The analysed dataset includes 384 degree values, corresponding to the number of nodes in the partition of the bi-partite network (Fig. 1a, green nodes) comprising reaction participants such as molecules, radicals, ions, etc. Fitting a power law to the observed degree distribution yields α = − 1.56 with standard error σ = 0.03. The cumulative probability of the node degree for the reaction components found in the system was assessed using a combination of significance value (p) and loglikelihood ratio (R) analyses to determine whether it more closely follows a heavy-tailed distribution, such as a power law, than a distribution with an exponential fall-off (Fig. 1b; please see “Methods” section for specific details of the statistical tools employed). The positive sign of log-likelihood ratio R = 198.3 and significance value of p < 0.05 (p = 2e−8) indicated that a heavy-tailed distribution fits the data better (see Refs.^63,97^ for more detailed discussion regarding the interpretation of R and p values). A similar comparison of heavy-tailed distribution types between power law and lognormal distributions (Fig. 1b) indicated that a lognormal distribution was a better fit than a power law distribution (negative sign of R = − 13.1, p = 2e−5).

### Enumerated closed-loop cycling of chemical species

Reactions that compose the network create at least 2618 possible closed cycles of molecules and photons. Chemical species ranked by frequency of presence within closed cycle subnetworks is provided in Supplementary Table S2. Water was explicitly excluded as an intermediate in enumerated cycles owing to its frequent presence as an unreactive bystander (solvent) molecule. Statistical analyses of cycle size distribution (number of nodes in each cycle, inclusive of participating chemical species and reactions) were conducted as described in the preceding paragraph and the Methods section. Each comparison had p values that exceeded a p < 0.05 threshold for statistically distinguishing between better fits^63^. This indicates that an exponential fall-off fit is not strongly distinguishable from heavy-tailed distributions such as power law (p = 0.90), lognormal (p = 0.37) or exponentially truncated power law (p = 0.29) fits (Fig. 1c).

**Figure 2:**
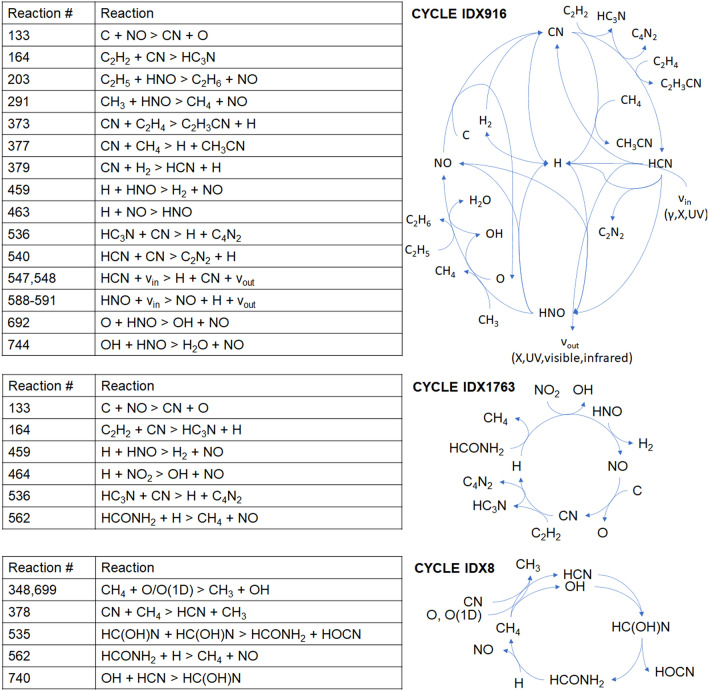
Three examples of simple cycles (IDX8, IDX1763 and IDX916) with net reactions that do not reduce to reactions present elsewhere within the radiolytic network. For simplicity, cycle depictions do not include stoichiometry.

Most cycles of reactions include low mass radicals directly produced from radiolysis and recombination of feedstock molecules (e.g., N_2_, CO_2_, H_2_O, etc.) such as H, N, O, OH, CN, NO, HNO, etc. H, OH and UV photons are each present in over 1000 of the total number of 2618 enumerated cycles. Three examples of overlapping cycles (IDX8, IDX1763, IDX916; IDX denotes the software-assigned cycle identification number) that include the most common species found in all cycles are illustrated in Fig. 2. The coupling of simple radicals to one another and to the initial substrates, and then to higher mass molecules produced over time, makes possible more complex cycles as molecular species diversify in the system. The cycle enumeration analysis indicates that as energy is input into the system, a facile return to the initial state (or to any particularly simple final state) becomes unlikely over time. As the number of organic species diversifies, and the total number of possible cyclical connections multiply accordingly, species that were not initially present within the system may persist through stability afforded by cyclic relationships^37,39^.

### Hierarchical stratification

**Figure 3:**
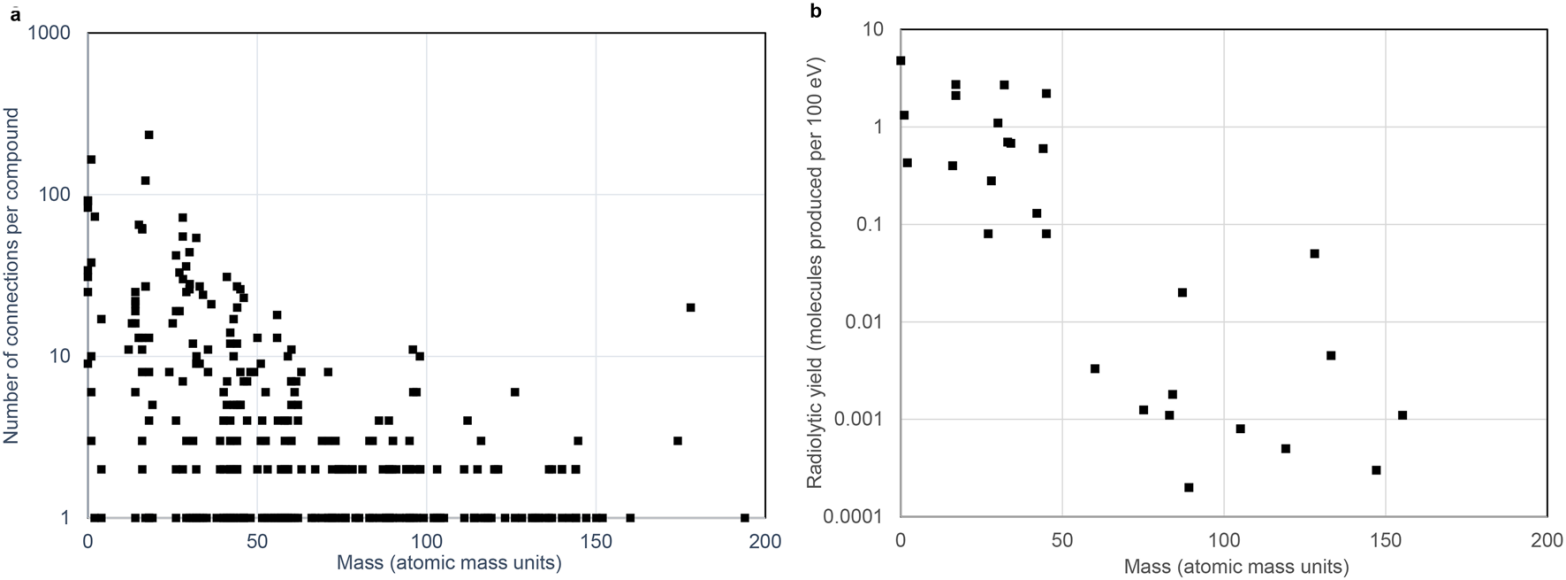
Indicators of hierarchical stratification in small molecule radiolytic chemical systems. (**a**) Connectivity degree per compound in the assembled radiolysis network, plotted versus chemical species mass. (**b**) Experimentally obtained radiolytic abundance (*G* values), plotted versus chemical species mass. Most plotted values are based on radiolysis of aqueous HCN/^-^CN. The plotted *G* value for hydrogen cyanide is based on irradiation of nitrogen and methane. See Supplementary Table S3 for all source value citations and accompanying notes.

A potential for hierarchical stratification within the radiolytic network is indicated by a histogram of compound connectivity plotted as a function of chemical species size (Fig. 3a). The connectivity distribution indicates that smaller-mass compounds are more frequently connected than larger compounds, and therefore that greater numbers of smaller-mass compounds should be produced and converted into other compounds at greater frequency within a radiolytic system than larger-mass compounds. However, these network description data lack estimates of relative product yield, or of the dominant synthetic pathways found in actual chemical systems where all of the reactions that make up the network are co-occurring with one another.

A more insightful physical parameter known as radiolytic yield (*G* value, typically reported in units of number of molecules produced per 100 eV of input absorbed; see notes in Supplemental Table S3) takes into account the complex balance of all production and degradation reactions that are co-occurring for each compound in a radiolysis experiment. *G* values can vary depending on conditions such as pH, temperature, presence of radical scavengers, and concentrations of starting materials or intermediate substrates formed. A species with a high *G* value (for a given array of experimental parameters) will be produced more frequently and probably be found in greater abundance than a species with a low *G* value. In experimental systems where radiolytic consumption outstrips production, *G* values can be negative. *G* values can therefore indicate dominant radiolytic pathways of consumption and production that tend to occur in actual chemical systems, and can be used to distinguish more frequently-occurring species and pathways from all possible such species and pathways mapped within the chemical reaction network. Radiolytic yield values depicted in this way reflect dominant modes of energy flow in irradiated chemical systems, analogous to the way time-integrated abundances of species offer a representation of energy flow in complex ecosystems that correlate with biomass or trophic level.

The plots of network connectivity distribution versus mass (Fig. 3a) and the radiolytic *G* values versus mass (Fig. 3b) depict a bottom-heavy energy-size spectrum of products where the time-averaged numbers of smaller objects greatly exceed those of larger objects. Bottom-heavy energy-size spectra are interpreted as indicators of hierarchical tiering and systemic stability, and are pervasive across ecosystem ecology (with a few, well-understood exceptions)^42^. The link between lower mass species heterogeneity leading to higher mass species heterogeneity, and the trend of decreasing abundance with increasing mass, implicates a relationship in which fewer, more massive molecules emerge from reactions between many, less massive input molecules and initial radicals. This hierarchical relationship is corroborated by direct observations from radiolytic experiments for which detailed quantitation data are available^2,48,54^ and the atmospheric chemistry of numerous solar system bodies^15^.

Robustness is a feature of living systems that is difficult to quantify compared to enumerable traits like number of cycles or connectivity distribution. Vector representations of graphs and relational structures, such as chemical reaction networks, enable the application of numerical data analysis to these complex structures^64^. We employed the representation learning framework *Node2vec*^65^ to conduct a pairwise comparison of the topological similarity of all reactions to one another, to assess whether disparate chemical reactions within the network occur in similar contexts; the identification of multiple, dissimilar reactions occurring in similar contexts would provide a measure of robustness. *Node2vec* has been shown to effectively learn features from diverse graph structures by employing a random walk exploration algorithm, which reduces connectivity paths around a node to vector representations^66–68^. The vector representations between any two reactions may be numerically compared to one another using cosine similarity, defined to equal the cosine of the angle between them, yielding an absolute value between 0 and 1 (indicating extrema of low and high similarity, respectively). Any two reactions in the assembled radiolysis network with a high cosine similarity (defined for this analysis as exceeding a numerical cut-off of 0.85) would be considered ‘synonymous’ with one another, in the sense of occurring within similar chemical contexts. The analysis uncovered 16 synonymous pairings with a cosine similarity of embedded reactions that exceeded the numerical cut-off (Supplementary Table S4). Excluding three placeholder equations (i.e., equations connecting known inputs and outputs but unknown intermediate species) and one mirrored forward/reverse reaction pairing, seven of these pairs involve radical species, and ten pairs involve either nitriles or carboxylic acids. This analysis suggests that nitriles and carboxylic acids form robust radical-driven subnetworks that connect geochemical substrates to organic compound production.

## Discussion

The topological attributes of this radiolytic network, i.e., (1) heterogeneous connectivity, (2) closed-loop cycling, and (3) hierarchy and a bottom-heavy energy-size spectrum, outline chemical conditions that have been sought for plausible prebiotic settings^13^. These attributes are intimately associated with generic capabilities of evolvability^27^ and self-organization^29^ that also underpin cell biology^69,70^ and ecosystem ecology. Two general observations are apparent based on the layout of the network. The first is that the subnetwork of radical interactions (Fig. 1a, shaded in red) dominates the core of the network and represents an efficient way to connect the production of different compounds to one another. The second is that water, beyond its long-recognized role as a solvent for prebiotic chemistry, can serve as an essentially inexhaustible source of powerful reagents that can drive reactions throughout the network. Water-derived radicals (namely, H/*e*_aq_^–^ and OH) are the most highly connected species, and their reactions yield some organic compounds in greater abundance than others of comparable carbon number or molecular mass. A notable example is simple hydroxyaldehyde production from hydrogen cyanide via Kiliani-Fischer homologation^49,50,71^, from which the synthesis of 2-aminooxazole^62^ and 2-aminoimidazole^72^ by the reaction of glycolaldehyde with cyanamide is afforded^48^.

A fit of the degree distribution of the reaction network participants to a power law yields an exponent value close to − 1.5, a value which is correlated with self-organizational behaviours in the vicinity of a phase transition between stable and chaotic states for many different complex systems such as earthquakes, avalanches, biological interactomes, neural activity, solar flares and cosmological structures^25,73–79^. Claims of ubiquitous power law scaling in biological and other complex networks^17,29,70^ have been reconsidered in light of more rigorous statistical treatments^80,81^; however, knowledge of whether a distribution is heavy-tailed or not is more important than whether it specifically fits a power law^82,83^. There is no universal prescription for the generative mechanisms that underlie such heavy-tailed distributions, nor for the possible self-organizational behaviours they are capable of exhibiting^82^; object properties that give rise to such distributions must be experimentally enumerated for each unique system^25^.

Radiolysis is generally considered to be a degradative chemical process, so how can it lead to the production and net accumulation of successively larger molecules? The patterns of compound synthesis observed in radiolytic systems may derive, in part, from the chemical kinetics of radicals. Lower mass radicals can more easily attain higher average speeds (which are proportional to m^‒0.5^) in the process of thermalization. These species may therefore be more likely to escape back-reaction than larger radicals at a given temperature. Small radicals may also yield progressively larger molecules over time because mon- and di-atomic species such as H, N, O, OH, CN, etc. possess relatively few internal degrees of freedom with which to distribute the energy released during radical recombination. This contrasts with larger molecules, where excess recombination energy can be quickly redistributed across many vibrational and rotational degrees of freedom^84^. This difference in numbers of degrees of freedom increases the probability that a recombination reaction that terminates a radical chain may lead to larger molecule production. It is important to note, however, that in the gas phase, excited CO and the CN radical have been observed to be exceptionally stable and tend to dominate initially, at least in the context of spark-discharge and laser-induced plasmas generated from a range of gaseous mixtures all containing sources of C, H, O and N^51^; differences between gas-phase and aqueous-phase radical reaction dynamics will be carefully considered in future network descriptions. The kinetic attributes of radicals, combined with their electrochemical extrema and ready sourcing from geochemical substrates, indicate that compound- and reaction-level radical attributes can play critical roles in producing complex network-level properties within abiotic chemical systems^15,85^. More specifically, the underlying requirements that can generate scale-free networks (continuous addition of new nodes, and increased likelihood of reactivity of new nodes with existing, highly-linked nodes)^28,86^ are consistent with the chemical properties of radicals such as H, OH, CN and the solvated electron.

The emergence of many closed cycles from only a handful of initial substrates is one of the most significant general attributes of radiolytic systems that finds parallels in the trophic dynamics of ecosystem networks^87^. Orgel^88^ observed: “The demonstration of the existence of a complex, nonenzymatic metabolic cycle, such as the reverse citric acid, would be a major step in research on the origin of life, while demonstration of an evolving family of such cycles would transform the subject…The recognition of sequences of plausible reactions that could close a cycle is an essential first step toward the discovery of new cycles, but experimental proof that such cycles are stable against the challenge of side reactions is even more important”. Families of radical cycles are known to play key roles in the atmospheric composition of different planetary bodies^89,90^, offering real examples that closed loops of radical reactions can be stable against side reactions. It remains to be seen whether radiolytically driven cycles meet the formal definition of autocatalytic sets as outlined by previous workers^91^, but they do implicate a potential for self-organization and dynamic feedback through cyclic ensembles prior to the emergence of catalytic macromolecules.

Alternative interpretations of the network topology stem from sources of potential observational bias. In some cases, the experiments that form the basis for the reaction network were designed to search for targeted species, or conducted with reference to specific type localities or prebiotic scenarios. In others, the analytical methods employed were limited to the technology available at the time, which improved in the years that followed. These potential sources of bias, though real, are nevertheless likely to exert a minimal impact on the observed network topology. The underlying experiments cover a wide range of parameters (i.e., total dose, dose rate, initial reactant selections and concentrations, energy sources) that span what would most likely be found in a natural system. Additionally, the chemical reactions inferred to be occurring in the experimental systems are evaluated within a context of accounting for the relative abundances of multiple products. By employing conservative criteria for network inclusion (Supplementary Information), further experimentation is likely to add to the existing network reactions, rather than alter or eliminate them in any statistically significant manner. Conservative criteria reduce the possibility that the inferred topological attributes described in this manuscript (connectivity distribution, hierarchical arrangement, etc.) will change as a result of greater experimental scrutiny. Finally, although this effort focused on the role that radicals play in shaping the network topology, future iterations should also evaluate the effects that ions may exert on expected network topology and attributes.

It remains an open question as to whether living systems can *only* emerge from non-living settings that already possess the network organizational features observed in current biology prior to the appearance of the first cell, or whether these attributes more likely arise as features at intermediate or even terminal points throughout a prebiotic chronology. The radiolytic network described here indicates that the network attributes that characterize forms of life on Earth and its ecologies may be tractably derived from abiotic network parallels found in relatively common atmospheric and geochemical conditions. Whether or not radiolysis was directly implicated in the historical origins of life on our planet requires more detailed study. From a broader perspective, though, the methods employed here to describe each of these reaction network attributes as parallels for higher-level ecosystem organization can be used to test diverse hypotheses about prebiotic chronologies such as the RNA World^92^, for evaluating the relative systemic complexities of terrestrial surface^93^ or hydrothermal^94^ chemosynthetic paradigms, or for testing theories about the likelihood of de novo emergence of pre-metabolic, autocatalytic cycles^88^. Considered separately from the question of life’s origins, this network may also serve as a basis for investigating whether radiolysis underpins a universal potential for organosynthetic self-organization or natural computation carried out within discrete chemical reaction networks^95^.

## Methods

### Chemical reaction network assembly

Chemical reaction network data are chemical reactions from radiolytic and polar chemical experiments that include key atomic species (CHONPS) of living systems. All reactions were assigned to chemical reaction categories (i.e., free radical reactions, physicochemical reactions, nitrile radical reactions, chloride reactions, etc.) based on their reactive substrates. Reactions that include solid, liquid and gaseous phases are combined into a single network, as it is assumed that (1) all phases and species within the ‘enclosed box’ of reactions can occur alongside one another; and (2) all reactants are within diffusive range of one another. These assumptions are consistent with plausible natural conditions that exist near a planetary surface:atmosphere interface, and with experimental radiolysis setups (many of which incorporate mixed phases) that form the basis of the compiled network.

A complete list of network reactions is provided as Supplementary Datafile S1 (Excel spreadsheet), and reaction criteria and source materials are described in the attached Supplementary Information file. Atoms are initially present in the system as their most geochemically abundant molecular species (H_2_O, CO_2_, N_2_, NaCl, a proxy for the apatite mineral group Ca_10_Cl_2_(PO_4_)_6_, and pyrite FeS_2_). The electromagnetic spectrum was binned into gamma, X-ray, UV, visible and infrared photons to account for reactions across key atomic and molecular energetic thresholds (i.e., strong nuclear binding force, inner valence electrons, outer valence electrons and ambient system temperature). Hydrated species complexed with water and reactions contingent upon an aqueous medium include water as a bystander species. Amino acid source polymers are chemically uncharacterised and are binned into polymer placeholder species that can be thermally hydrolysed to yield amino acids. Reaction rates and kinetic coefficients were omitted from this study.

The energy sources that could drive network structure and chemosynthesis were forms of radiation sourced from radiogenic uranium oxide. Uranium is a geochemical proxy for naturally-occurring energy sources such as galactic cosmic radiation^58^, solar flares^59^ and planetary radiation belts^60^. The assembled database includes 782 reactions and 386 distinct atomic, molecular, and photonic species.

### Chemical reaction network visualisation

The acquired reaction equations were used to construct a reaction network where nodes are divided into two sets, i.e. partitions, and connections are allowed only between nodes in different partitions. Networks of this type are called bi-partite networks. The first partition in the constructed network includes reaction participants, such as atomic, molecular, and photonic species; the second partition includes reactions themselves. The network is constructed as a directed network where directionality reflects the form of the stoichiometric equation: the entities on the left-hand side of the equation are connected to the node representing the reaction, and the reaction node is connected to the entities on the right-hand side of the equation. This definition of directionality simplifies network analytics, such as cycle search. It is related to, but not definitive of, reversibility of the chemical reactions. The resulting network was graphed and analysed using Gephi 0.9.2^96^. The network file is included as Datafile S2 (.graphml format).

### Statistical analyses

Analysis of the degree distribution was performed following the methodology introduced and extensively discussed in Abbot et al. using a python library *powerlaw*^97^. A log-likelihood test is employed to evaluate pairs of alternative distributions as possible sources of the observed data. For example, the test elucidates whether the dataset exhibits a heavy-tailed distribution by comparing a power law fit (the simplest approximation of a heavy-tailed distribution on a finite interval) to one with an exponential fall-off. The test also helps to check if alternative distributions, such as a lognormal distribution, exponentially truncated power law, etc. offer better fits than a power law. Per convention used in *powerlaw* library implementation, the computed loglikelihood ratio *R* for the pair of distributions *(d1, d2)* is positive if data are more likely to fit the first distribution *d1*, and negative if the data are more likely to fit the second distribution *d2*. Loglikelihood ratio is accompanied by the significance value *p;* following Clauset et al., we consider *p* > *0.*05 as an indicator that neither distribution is a stronger fit^63^. Detailed discussion of these tools is beyond the scope of our contribution; for more details, we refer the inquisitive reader to the original papers describing these tools and implementation^63,97^.

### Closed loop cycle enumeration

Closed loops of chemical species in the directed bi-partite network were identified through exhaustive enumeration between pairs of nodes by finding the shortest paths from the first node to the second and all the shortest paths from the second node to the first. Pair-wise combinations of the forward/backward paths would constitute distinguishable cycles. Enumerated cycles were expressed as sub-networks formed by these forward–backward paths; each sub-network was labelled with an ordered sequence of the indices of the included nodes to identify unique subnetworks. Water, as a frequent bystander molecule, was omitted as a possible link to prevent overcounting of trivial closed cycles. All cycles were assigned a numerical identifier according to the order of discovery within the network, with a prefix IDX. The order of discovery within the network, and thus the enumeration of the cycles, carries no particular physical or chemical significance. Each IDX cycle was saved as an individual Graphml network file, which are available upon request. Summary information about all of the IDX cycles (species composition of the cycles and cycle size) are included as Supplementary Datafile S3 (Excel spreadsheet).

### Assessing systemic robustness with a machine-learning algorithm

The framework for representation learning on graphs *Node2vec*^65^ was used to learn continuous features of molecules and reactions, and assess whether disparate chemical reactions within the network occur within similar contexts regardless of their reaction category assignments. Network-based chemical semantic similarity was evaluated by carrying out random walks within the network, embedding the nodes visited by each walk into a vector space, and evaluating cosine similarity of the embedded reactions with a cut-off for similar reactions set at 0.85.

## Supplementary Information

Supplementary Information 1.

Supplementary Information 2.

Supplementary Information 3.

Supplementary Information 4.

## Data Availability

Data files generated and analysed during the current study, but not included as supplementary information files, are available from the corresponding author on request.
