# Supplementary material for: Radiolysis generates a complex organosynthetic chemical network
Source: Sci Rep. 2021 Jan 18;11:1743. doi: 10.1038/s41598-021-81293-6 (PMC7813863; doi:10.1038/s41598-021-81293-6)
Supplement: Supplementary file 4 — Supplementary Information 4. [file 41598_2021_81293_MOESM4_ESM.pdf]

Radiolysis Generates a Complex Organosynthetic Chemical Network

Zachary R. Adam, Albert C. Fahrenbach, Sofia Marie Jacobson, Betul Kacar and Dmitry Yu. Zubarev

Correspondence regarding supplementary data files may be directed to: zadam@arizona.edu

SUPPLEMENTAL INFORMATION

Chemical Network Source Material and Selection Criteria

Chemical reaction network distribution data were collected by collating chemical reactions from radiolytic and polar chemical research findings that include all of the key atomic species of living systems (CHONPS, along with Fe which is a requisite metal cofactor for the electron transport chain, and sea salt NaCl)[1-44].

We built a corpus of 44 papers; the papers were selected according to the domain knowledge of the authors and expanded via cross-referencing. The corpus contains peer-reviewed publications in English, spanning a period of time from 1961 to 2020, and 31 journals. The following criteria were used to assign a reaction within the network based on the reported results:

1. Evidence for radical products had to be directly observed in presented data (GCMS, LCMS, NMR, etc.), and identified using standard NIST chemical database signatures or by comparison with verified, off-the-shelf feedstocks.
2. Unobserved intermediates had to reasonably account for the presence of observed products (ideally, multiple products), or be consistent with model calculations reported by other workers.
3. Non-radical equations had to have been observed and reported in peer-reviewed manuscripts, or consist of common chemical knowledge.

A complete list of network reactions and cycles is included as a supplemental Excel Spreadsheet data file, and a complete list of referenced source materials is provided below.

Supplementary Table S1. A list of the most connected species within the radiolytic network.

Chemical Object	In degree	Out degree	Total Degree
H_2O	86	148	234
H	98	67	165
OH	53	69	122
Gamma rays	3	89	92
Infrared photons	61	31	92
X rays	40	51	91
UV photons	41	42	83
H_2	57	16	73
CO	60	12	72
CH_3	31	34	65
O	30	32	62
CH_4	22	39	61
N_2	16	39	55
O_2	24	30	54
NO	30	14	44
C_2H_2	26	16	42
H(+)	19	19	38
HCO	20	16	36
Beta rays	30	4	34
HCN	14	19	33
CH_3CN	5	26	31
e(-)	20	11	31
C_2H_4	12	18	30
C_2H_6	8	20	28
CO_2	14	13	27
HO_2	11	16	27
NH_3	16	11	27
OH(-)	24	3	27
H_2CO	12	14	26
$HCONH_2$	7	19	26
(3)CH_2	12	13	25
C_2H_5	10	15	25
e_{aq}(-)	6	19	25
H_2O_2	4	20	24
NO_2	13	10	23
(1)CH_2	13	9	22
HCl	7	14	21
N	12	9	21
CH_3CHO	8	12	20
H_4P_2O7	10	10	20
(4S)N	10	9	19
C_2H_3	5	14	19
CN	5	14	19

Supplementary Table S2. A list of the most frequently occurring chemical species in enumerated closed loops of reactions within the network, excluding water due to its prominent role as a solvent medium.

Compound Name	Number of cycles
H	1570
OH	1032
UV photons	1021
X-rays	966
H_2	746
CH_3	711
C_2H_2	593
CH_4	551
O	470
CO	386
N_2	381
HCN	359
CO_2	357
O_2	333
(3)CH_2	326
C_2H_5	320
NO	312
H_2CO	296
C_2H_4	292
$HCONH_2$	283
HCO	280
C_2H_6	253
H(+)	245
(4S)N	215
CH	201
CN	192
CH_2CO	171
C_2H	167
C_2H_3	158
Infrared photons	156
(2D)N	150
HO_2	144
HPO_4(2-)	135
NH	135
NH_3	134

Supplementary Table S3. Source data and explanatory notes for representative G values depicted in Figure 3.

The radiolytic yield, often referred to as the '*G* value', refers to the number of molecules of reactant consumed or product formed per 100 eV of energy absorbed. In a system irradiated by incident particles with energies that greatly surpass the typical bond energies between atoms in molecules (~1-10 eV), nearly all compounds present in the system are expected to be both produced and degraded by that energy source. There are multiple possible reactions and pathways that can affect the balance of whether a compound is more produced or more degraded over time, and it is often difficult to predict at the outset which of these possible reactions will be dominant. A compound's *G* value is therefore measured as a parameter that approximates the balance of productivity of a compound when the details of exactly how that compound is formed and degraded are difficult or impossible to predict with accuracy.

G values are therefore informative, but not necessarily predictive, of systemic behavior for a given compound in a radiolytic system. Yield can vary if different reactants are employed, or if conditions such as temperature or pH are varied. Accurate quantification of product yield can be a laborious process in radiolytic chemistry experimentation, and for this reason, *G* values are not reported for all compounds confirmed through chemical analysis. This is why the chemical reaction database includes many more compounds than a plot such as Figure 3 based on reported *G* values. Despite these caveats, *G* values nevertheless indicate the broad contours of energy flow through a complex radiolytic system, since they tend not to vary by more than an order of magnitude for a given compound with a given reactant substrate.

In addition to the three criteria described above for reactions that were incorporated into the chemical reaction network, the following criteria were used to determine which *G* values would be incorporated into Figure 3:

1. *G* values were used from prebiotically relevant compounds (amino acids and nucleotide precursors) and the intermediates that most directly link these compounds to plausible geochemical substrates such as N_2, H_2O and dissolved salts.
2. *G* values are plotted as reported from manuscripts spanning 60 years of radiolysis research; many reported values do not include error estimates, and different analytical methods have been employed over this time period, so values are plotted as points without further elaboration (i.e., without error bars or statistical variability).
3. Changes in the selection of initial substrates (e.g., formate versus formic acid versus formaldehyde) and their concentrations can vary *G* values, so we have included only those substrates derived from radiolysis of C1 compounds (preferably HCN for consistency, since this is the most highly studied substrate for which *G* values of prebiotically relevant compounds are reported) or common geochemical substrates.
4. In cases where *G* values are reported for many different pH values, we have plotted a value corresponding to nearly neutral pH.
5. In cases where *G* values for many concentrations of initial substrates are reported for a compound, we have plotted a reported value near the mean concentration.
6. In cases where multiple G values are reported from different studies or workers, we have plotted a value that is best supported by experimental observations rather than models;

which uses substrates with more abundant naturally-occurring analogs; and by workers with a published history of reliable and replicable results.

7. When multiple values with different experimental parameters have been reported for a given compound, the list of substrates and sources below includes all manuscripts reviewed (even if they were not selected for the plot) to provide a supporting record that reported values may differ slightly but are nevertheless comparable to one another.

The following table describes the sources and details of each of the plotted G values that compose Figure 3, ranked in order of mass from largest to smallest.

**Species**	**Mass (amu)**	**G value (#/100 eV)**	**Substrates**	**Sources**
Histidine	155.2	1.10E-03	HCN,H_2O	Draganic et al., 1976[45]
Glutamic acid	147.1	3.00E-04	HCN,H_2O	Draganic et al., 1976[45]
Aspartic acid	133.1	4.50E-03	HCN,H_2O	Draganic et al., 1976[45]
Oxalate	128	0.05	$(NH_4)HCO_3$,H_2O	Draganic et al., 1991[19]
Threonine	119.1	5.00E-04	HCN,H_2O	Draganic et al., 1976[45]
Serine	105.1	8.00E-04	HCN,H_2O	Draganic et al., 1976[45]
Alanine	89.1	2.00E-04	HCN,H_2O	Draganic et al., 1976[45]
Glyceronitrile	87.08	0.02	HCN, H_2O, NaCl, KCN, NaCN	Yi et al., 2020[44]
2-Aminoimidazole	83.092	1.1E-3	HCN, H_2O, NaCl, KCN, NaCN	Yi et al., 2020[44]
2-Aminooxazole	84.077	1.8E-03	HCN, H_2O, NaCl, KCN, NaCN	Yi et al., 2020[44]
Glycine	75.1	1.25E-03	HCN,H_2O	Draganic et al., 1976[45]
Glycolaldehyde	60.052	0.0033	HCN, H_2O, NaCl, KCN, NaCN; CH_3CN	Yi et al., 2020[44]; Draganic et al., 1980[7]
Formamide	45	0.08	HCN, CH_3CN, H_2O	Adam et al., 2018[46]
Formate	45	2.2	$(NH_4)HCO_3$, H_2O	Draganic et al., 1991[19]
CO_2	44	0.60	HCN, H_2O	Draganic et al., 1976[45]; Ogura et al., 1972[47]
Cyanamide	42.04	0.13	HCN, H_2O, NaCl, KCN, NaCN	Yi et al., 2020[44]; Yi et al., 2018[48]
H_2O_2	34	0.68	H_2O	Yamaguchi et al., 2005[49]; Draganic et al., 1969[50]; Elliot et al., 1990[51]
NH_2OH	33	0.7	NH_3, H_2O	Pagsberg, 1972[52]
N_2H_2	32	2.7	NH_3, H_2O	Pagsberg, 1972[52]
HCHO (CH_2O)	30	1.1	HCN, H_2O, CH_3OH	Draganic et al., 1976[45]; Ogura et al., 1972[47]; Nuclear Science Abstracts, Volume 18, Issues 13-19, p. 4509[53]; Seki et al., 1968[54]

				Meshitsuka and Burton, 1958[55]
CO	28	0.28	H_2O, HCOOH; CH_3OH	Adams and Hart, 1962[56]; Meshitsuka and Burton, 1958[55]
HCN	27	0.08	N_2, CH_4; N_2, C_2H_4	Siderer and Sato, 1975[57]; Oka et al., 1968[58]
OH	17	2.72	H_2O	Yamaguchi et al., 2005[49]; Draganic et al., 1969[50]; Draganic et al., 1973[59]; Elliot et al., 1990[51]
NH_3	17	2.10	HCN, H_2O; NH_2CN, H2O; N_2, CH_4; N_2, H_2O, CH_3OH, HCl	Draganic et al., 1976[45]; Draganic et al., 1978[60]; Siderer and Sato, 1975[57]; Ogura et al., 1972[47]; Getoff, 1966[61];
CH_4	16	0.4	CH_3OH	Nuclear Science Abstracts, Volume 18, Issues 13-19, p. 4509[53]; Meshitsuka and Burton, 1958[55]
H_2	2	0.43	H_2O; CH_3OH	Yamaguchi et al., 2005[49]; Draganic et al., 1969[50]; Meshitsuka and Burton, 1958[55]
H	1	1.32	H_2O	Yamaguchi et al., 2005[49]; Elliot et al., 1990[51]
Solvated/hydrated electron	0.0005	4.78	H_2O	Yamaguchi et al., 2005[49]; Draganic et al., 1973[59]; Elliot et al., 1990[51]

Supplementary Table 4. Complete list of all semantically similar chemical reactions detected within the radiolytic network using the *Node2vec* machine-learning algorithm. Evaluations are based on continuous embedding of network nodes obtained using *Node2vec* framework for representation learning on graphs, with a cutoff set for similarity indices greater than 0.85. Radical species are indicated in red. Placeholder equations (where observations are well-supported but the details of the intermediate steps linking inputs to products are unknown or uncharacterized) are indicated in blue.

Reaction Number	Reaction Category	Reaction	Synonymy Index
458	Carboxylic Acid	$H + HCOOH > H_2 + COOH$	0.8851
447	Radical Reactions	$H + COOH > HCOOH$	
276	Carboxylic Acid	$CH_2COOH + COOH > HOOCCH_2COOH$	0.8630
415	Radical Reactions	$COOH + CHOOH > CH_2(OH)COOH$	
403	Carboxylic Acid	$CO_3(-) + HCOO(-) > HCO_3(-) + COO(-)$	0.9023
413	Radical Reactions	$COO(-) + HCO_3(-) > HCOO(-) + CO_3(-)$	
55	Carboxylic Acid	$2\ CH_2COOH > HOOC(CH_2)_2COOH$	0.9639
275	Radical Reactions	$CH_2COOH + CH_2COOH > (CH_2COOH)_2$	
411	Carboxylic	$COO(-) + H_2O > HOCOO(2-) + H(+)$	0.8899
416	Radical Reactions	$COOH > COO(-) + H(+)$	
707	Carboxylic	$O_2 + CH_2COO(-) > O_2CH_2COO(-)$	0.8689
721	Carboxylic	$O_2CH_2COO(-) + O_2CH_2COO(-) > CHOCOOH + H_2CO + CO_2$	
722	Carboxylic	$O_2CH_2COOH + O_2CH_2COOH > CH_2OHCOOH + CHOCOOH + H_2CO + CO_2$	0.8868
720	Carboxylic	$O_2CH_2COO(-) + H(+) > O_2CH_2COOH$	
247	Carboxylic	$C_4H_4N_2O_2 + O_2 + Fe(2+) > CH_4N_2O + HOOCCHCHCOOH$	0.9165
246	Carboxylic	$C_4H_4N_2O_2 + H_2O_2 + Fe(2+) > CH_4N_2O + HOOCCHCHCOOH$	
662	RNA Nucleotide	$NH_2CN + H_2O > CH_4N_2O$	0.9676
253	Radical Reactions	$C_5H_6N_2O_2 + \gamma + H_2O > CH_4N_2O$	
662	RNA Nucleotide	$NH_2CN + H_2O > CH_4N_2O$	0.8583
361	Radical Reactions	$CH_4N_2O + \gamma + H_2O > C_2H_2O_4$	
360	RNA Nucleotide	$CH_4N_2O + C_3H_3NO > C_4H_5N_3O + H_2O$	0.8677
253	Radical Reactions	$C_5H_6N_2O_2 + \gamma + H_2O > CH_4N_2O$	
662	RNA Nucleotide	$NH_2CN + H_2O > CH_4N_2O$	0.8610
360	RNA Nucleotide	$CH_4N_2O + C_3H_3NO > C_4H_5N_3O + H_2O$	
454	Nitrile	$H + HCN > H_2CN$	0.8951
476	Radical Reactions	$H_2CN + H > HCN + H_2$	
478	Nitrile	$H_2CNH + HCN > NC\text{-}CH_2\text{-}NH_2$	0.8578
477	Nitrile	$H_2CN + H_2CN > HCN + H_2CNH$	
535	Nitrile	$HC(OH)N + HC(OH)N > HCONH_2 + HOCN$	0.9329
740	Nitrile	$OH + HCN > HC(OH)N$	
480	Nitrile	$H_2CO + HCN > NC\text{-}CH_2\text{-}OH$	0.9315
0	Nitrile	$2HNC\text{-}CH_2\text{-}OH > NC\text{-}CH_2\text{-}OH + H_2NC\text{-}CH_2\text{-}OH$	

OTHER SUPPLEMENTARY DATAFILES

Data file S1. Chemical Reaction Network Database (Excel Spreadsheet)

Data file S2. Reaction Network File (Graphml)

Data file S3. Enumerated Cycle Summary File (Excel Spreadsheet)

SUPPLEMENTARY INFORMATION REFERENCES

1 Lousada, C. M., Johansson, A. J., Brinck, T. & Jonsson, M. Mechanism of H2O2 decomposition on transition metal oxide surfaces. *The Journal of Physical Chemistry C* **116**, 9533-9543 (2012).

2 Butarbutar, S., Guzonas, D., Stuart, C., Meesungnoen, J. & Jay-Gerin, J.-P. Density dependence of the radiolysis yields of primary species from fast neutron-irradiated supercritical water at 400 o C. (Atomic Energy of Canada Limited, 2014).

3 Zahnle, K. J. Photochemistry of methane and the formation of hydrocyanic acid (HCN) in the Earth's early atmosphere. *Journal of Geophysical Research: Atmospheres* **91**, 2819-2834 (1986).

4 Lara, L. M., Lellouch, E., López-Moreno, J. & Rodrigo, R. Vertical distribution of Titan's atmospheric neutral constituents. *Journal of Geophysical Research: Planets* **101**, 23261-23283 (1996).

5 Macdonald, R. G. & Miller, O. A. Low dose-rate radiolysis of nitrogen: yield of nitrogen atoms, N (4S) and N (2D, 2P). *Radiation Physics and Chemistry (1977)* **26**, 63-72 (1985).

6 Balucani, N. *et al.* Cyanomethylene formation from the reaction of excited nitrogen atoms with acetylene: a crossed beam and ab initio study. *Journal of the American Chemical Society* **122**, 4443-4450 (2000).

7 Draganić, I., Jovanović, S., Niketić, V. & Draganić, Z. The radiolysis of aqueous acetonitrile: compounds of interest to chemical evolution studies. *Journal of molecular evolution* **15**, 261-275 (1980).

8 Dey, G. Nitrogen compounds' formation in aqueous solutions under high ionizing radiation: An overview. *Radiation Physics and Chemistry* **80**, 394-402 (2011).

9 Koyano, I., Tanaka, I. & Omura, I. Chemi-Ionization in Photoexcited Acetylene. *The Journal of Chemical Physics* **40**, 2734-2735 (1964).

10 Zhou, L., Maity, S., Abplanalp, M., Turner, A. & Kaiser, R. I. On the radiolysis of ethylene ices by energetic electrons and implications to the extraterrestrial hydrocarbon chemistry. *The Astrophysical Journal* **790**, 38 (2014).

11 Hayon, E. & Nakashima, M. Intermediates produced in the flash photolysis of acetone and amides in aqueous solution. *The Journal of Physical Chemistry* **75**, 1910-1914 (1971).

12 Infante, G. A., Jirathana, P., Fendler, J. H. & Fendler, E. J. Radiolysis of pyrimidines in aqueous solutions. Part 1.—Product formation in the interaction of e–aq,· H,· OH and Cl–2· with thymine. *Journal of the Chemical Society, Faraday Transactions 1: Physical Chemistry in Condensed Phases* **69**, 1586-1596 (1973).

13 Josimović, L. & Draganić, I. The radiolysis of acetic acid in aqueous solutions and acetic acid-water mixtures. *International Journal for Radiation Physics and Chemistry* **5**, 505-512 (1973).

14 Stevens, G., Clarke, R. M. & Hart, E. J. Radiolysis of aqueous methane solutions. *The Journal of Physical Chemistry* **76**, 3863-3867 (1972).

15 Hayon, E., Ibata, T., Lichtin, N. & Simic, M. Sites of attack of hydroxyl radicals on amides in aqueous solution. *Journal of the American Chemical Society* **92**, 3898-3903 (1970).
16 Adam, Z. R. *et al.* Estimating the capacity for production of formamide by radioactive minerals on the prebiotic Earth. *Scientific reports* **8**, 265 (2018).
17 Navarro-Gonzalez, R., Negron-Mendoza, A. & Chacon, E. The γ-irradiation of aqueous solutions of urea. Implications for chemical evolution. *Origins of Life and Evolution of the Biosphere* **19**, 109-118 (1989).
18 Buxton, G. & Sellers, R. Acid dissociation constant of the carboxyl radical. Pulse radiolysis studies of aqueous solutions of formic acid and sodium formate. *Journal of the Chemical Society, Faraday Transactions 1: Physical Chemistry in Condensed Phases* **69**, 555-559 (1973).
19 Draganić, Z. D. *et al.* Radiolysis of aqueous solutions of ammonium bicarbonate over a large dose range. *International Journal of Radiation Applications and Instrumentation. Part C. Radiation Physics and Chemistry* **38**, 317-321 (1991).
20 Jiang, P.-Y. *et al.* Pulse radiolysis study of concentrated phosphoric acid solutions. *Journal of the Chemical Society, Faraday Transactions* **88**, 3319-3322 (1992).
21 Friedmann, N. & Miller, S. L. Synthesis of valine and isoleucine in primitive earth conditions. *Nature* **221**, 1152 (1969).
22 Oró, J. & Kamat, S. Amino-acid synthesis from hydrogen cyanide under possible primitive earth conditions. *Nature* **190**, 442 (1961).
23 Takahashi, J.-i. *et al.* Photochemical abiotic synthesis of amino-acid precursors from simulated planetary atmospheres by vacuum ultraviolet light. *Journal of applied physics* **98**, 024907 (2005).
24 Kobayashi, K. *et al.* Formation of bioorganic compounds in simulated planetary atmospheres by high energy particles or photons. *Advances in Space Research* **27**, 207-215 (2001).
25 Gautier, T. *et al.* Nitrile gas chemistry in Titan's atmosphere. *Icarus* **213**, 625-635 (2011).
26 Horne, G. P., Donoclift, T. A., Sims, H. E., Orr, R. M. & Pimblott, S. M. Multi-scale modeling of the gamma radiolysis of nitrate solutions. *The Journal of Physical Chemistry B* **120**, 11781-11789 (2016).
27 Takano, Y., Masuda, H., Kaneko, T. & Kobayashi, K. Formation of amino acids from possible interstellar media by γ-rays and UV irradiation. *Chemistry letters* **31**, 986-987 (2002).
28 Fahrenbach, A. C. *et al.* Common and potentially prebiotic origin for precursors of nucleotide synthesis and activation. *Journal of the American Chemical Society* **139**, 8780-8783 (2017).
29 You, J., Ying, R., Ren, X., Hamilton, W. L. & Leskovec, J. Graphrnn: Generating realistic graphs with deep auto-regressive models. *arXiv preprint arXiv:1802.08773* (2018).
30 Saladino, R., Crestini, C., Ciciriello, F., Costanzo, G. & Di Mauro, E. About a formamide-based origin of informational polymers: syntheses of nucleobases and favourable thermodynamic niches for early polymers. *Origins of Life and Evolution of Biospheres* **36**, 523-531 (2006).
31 Kitadai, N. & Maruyama, S. Origins of building blocks of life: A review. *Geoscience Frontiers* **9**, 1117-1153 (2018).
32 Powner, M. W., Gerland, B. & Sutherland, J. D. Synthesis of activated pyrimidine ribonucleotides in prebiotically plausible conditions. *Nature* **459**, 239 (2009).
33 Draganic, I., Draganic, Z., Petkovic, L. & Nikolic, A. Radiation chemistry of aqueous solutions of simple RCN [hydrogen or alkyl cyanide] compounds. *Journal of the American Chemical Society* **95**, 7193-7199 (1973).
34 Bielski, B. & Allen, A. O. Radiation chemistry of aqueous cyanide ion. *Journal of the American Chemical Society* **99**, 5931-5934 (1977).

35 Moutou, G. *et al.* Equilibrium of α-aminoacetonitrile formation from formaldehyde, hydrogen cyanide and ammonia in aqueous solution: Industrial and prebiotic significance. *Journal of Physical Organic Chemistry* **8**, 721-730 (1995).
36 Yu, X.-Y. & Barker, J. R. Hydrogen peroxide photolysis in acidic aqueous solutions containing chloride ions. I. Chemical mechanism. *The Journal of Physical Chemistry A* **107**, 1313-1324 (2003).
37 El Omar, A. K., Schmidhammer, U., Rousseau, B., LaVerne, J. & Mostafavi, M. Competition Reactions of H2O•+ Radical in Concentrated Cl–Aqueous Solutions: Picosecond Pulse Radiolysis Study. *The Journal of Physical Chemistry A* **116**, 11509-11518 (2012).
38 Balcerzyk, A. *et al.* Picosecond pulse radiolysis of direct and indirect radiolytic effects in highly concentrated halide aqueous solutions. *The Journal of Physical Chemistry A* **115**, 9151-9159 (2011).
39 Wang, T. X. & Margerum, D. W. Kinetics of reversible chlorine hydrolysis: temperature dependence and general-acid/base-assisted mechanisms. *Inorganic Chemistry* **33**, 1050-1055 (1994).
40 Gerritsen, C. M. & Margerum, D. W. Non-metal redox kinetics: hypochlorite and hypochlorous acid reactions with cyanide. *inorganic Chemistry* **29**, 2757-2762 (1990).
41 Schurter, L. M., Bachelor, P. P. & Margerum, D. W. Nonmetal redox kinetics: mono-, di-, and trichloramine reactions with cyanide ion. *Environmental science & technology* **29**, 1127-1134 (1995).
42 Obodovskiy, I. *Radiation: Fundamentals, Applications, Risks, and Safety*. (Elsevier, 2019).
43 Eisenberger, P. & Platzman, P. Compton scattering of x rays from bound electrons. *Physical Review A* **2**, 415 (1970).
44 Yi, R. *et al.* A Continuous Reaction Network That Produces RNA Precursors. *Proceedings of the National Academy of Sciences*, doi:10.1073/pnas.1922139117 (2020).
45 Draganić, Z., Draganić, I. & Borovičanin, M. The radiation chemistry of aqueous solutions of hydrogen cyanide in the megarad dose range. *Radiation research* **66**, 42-53 (1976).
46 Adam, Z. R. *et al.* Estimating the capacity for production of formamide by radioactive minerals on the prebiotic Earth. *Scientific reports* **8**, 1-8 (2018).
47 Ogura, H., Fujimura, T., Murozono, S., Hirano, K. & Kondo, M. Radiolysis of Hydrogen Cyanide in an Aqueous System,(III). *Journal of Nuclear Science and Technology* **9**, 339-343 (1972).
48 Yi, R., Hongo, Y., Yoda, I., Adam, Z. R. & Fahrenbach, A. C. Radiolytic synthesis of cyanogen chloride, cyanamide and simple sugar precursors. *ChemistrySelect* **3**, 10169-10174 (2018).
49 Yamaguchi, H., Uchihori, Y., Yasuda, N., Takada, M. & Kitamura, H. Estimation of yields of OH radicals in water irradiated by ionizing radiation. *Journal of radiation research* **46**, 333-341 (2005).
50 Draganic, I., Nenadovic, M. & Draganic, Z. D. Radiolysis of HCOOH+ O2 at pH 1.3-13 and the yields of primary products in. gamma. radiolysis of water. *The Journal of Physical Chemistry* **73**, 2564-2571 (1969).
51 Elliot, A. J., Chenier, M. P. & Ouellette, D. C. g-Values for γ-irradiated water as a function of temperature. *Canadian Journal of Chemistry* **68**, 712-719 (1990).
52 Pagsberg, P. B. Investigation of the NH2 radical produced by pulse radiolysis of ammonia in aqueous solution. *Aspects of Research at Risø* **5**, 209 (1972).
53 Norris, J. W. Vol. 18 4509 (1964).

54 Seki, H., Nagai, R. & Imamura, M. γ-Radiolysis of a Binary Mixture of Methanol and Water. The Formation of Formaldehyde in the Radiolysis of Liquid Methanol. *Bulletin of the Chemical Society of Japan* **41**, 2877-2881 (1968).
55 Meshitsuka, G. & Burton, M. Radiolysis of liquid methanol by Co60 gamma-radiation. *Radiation research* **8**, 285-297 (1958).
56 Adams, G. & Hart, E. J. Radiolysis and photolysis of aqueous formic acid. Carbon monoxide formation. *Journal of the American Chemical Society* **84**, 3994-3999 (1962).
57 Siderer, Y. & Sato, S. The γ-radiolysis of liquid nitrogen containing methane. *Bulletin of the Chemical Society of Japan* **48**, 2383-2384 (1975).
58 Oka, T., Kato, R., Sato, S. & Shida, S. Hydrogen Cyanide Formation in the Gas-phase Radiolysis of Mixtures of Nitrogen and Ethylene. *Bulletin of the Chemical Society of Japan* **41**, 2192-2193 (1968).
59 Draganic, Z. & Draganic, I. Formation of primary yields of hydroxyl radical and hydrated electron in the. gamma.-radiolysis of water. *The Journal of Physical Chemistry* **77**, 765-772 (1973).
60 Draganić, Z., Draganić, I. & Jovanović, S. The radiation chemistry of aqueous solutions of cyanamide. *Radiation Research* **75**, 508-518 (1978).
61 Getoff, N. Radiation-induced synthesis of ammonia from nitrogen and water. *Nature* **210**, 940-941 (1966).
